# COVID-19 Convalescent Plasma Therapy: Long-term Implications

**DOI:** 10.1093/ofid/ofad686

**Published:** 2023-12-29

**Authors:** Hyunah Yoon, Yi Li, Keith S Goldfeld, Gia F Cobb, Caroline L Sturm-Reganato, Luis Ostrosky-Zeichner, Dushyantha T Jayaweera, Julie V Philley, Mahalia S Desruisseaux, Marla J Keller, Judith S Hochman, Liise-anne Pirofski, Mila B Ortigoza, for the CONTAIN-Extend Study Group, Judith S Hochman, Bruce N Cronstein, Deborah Keeling, Norka Rappoport, Jenna Saraga, James Holahan, Mila B Ortigoza, Liise-anne Pirofski, Hyunah Yoon, Caroline L Sturm-Reganato, Gia F Cobb, Rakshit Andela, Yousef Darwish, Monica R Taveras, Patrick S Xin, Jeff LaFleur, Levi Cleare, Keith S Goldfeld, Yi Li, Mila B Ortigoza, Mary L O'Keeffe, Gia F Cobb, Caroline L Sturm-Reganato, Fatema Z Rahman, Adeyinka O Ajayi, Sara L Rodriguez, Eduardo Iturrate, Jacqueline M Gallagher, Ololade E Thomas, Danibel Ramos, Charlotte C Fong, Liise-anne Pirofski, Hyunah Yoon, Marla J Keller, Andrea A Asencio, Isaiah Eke, James Castro, Jidong Shan, Alex Chalco, Jeff LaFleur, Levi Cleare, Mahalia Desruisseaux, Grace M Cortezzo, Erica Rocco, Oscar Bate Akide Ndunge, Catherine Parmelee, Gina Solomon, Staci Cahil, Dushyantha T Jayaweera, Chin Chin Lee, Daru L Ransford, Deniz Dasmany, Andres Corona, Kenia Moreno, Gledys L Martinez, Christopher Otero, David D McPherson, Luis Ostrosky-Zeichner, Bela Patel, Masayuki Nigo, Ryan M Huebinger, Goutham Dronavalli, Carolyn Z Grimes, Virginia E Umana, Maria D Hernandez, Laura E Nielsen, Taylor P Stutz, Mehriban Mammadova, Andrew N Dentino, Timothy R Heath, Jessica G Martin, Fatimah O Bello, Erik Hinojosa, Julie V Philley, Megan S Devine, Rebekah L Hibbard, Anne M Ford

**Affiliations:** Division of Infectious Diseases, Department of Medicine, Albert Einstein College of Medicine, Montefiore Medical Center, Bronx, New York, USA; Division of Biostatistics, Department of Population Health, NYU Grossman School of Medicine, New York, New York, USA; Division of Biostatistics, Department of Population Health, NYU Grossman School of Medicine, New York, New York, USA; Department of Medicine, NYU Grossman School of Medicine, New York, New York, USA; Department of Medicine, NYU Grossman School of Medicine, New York, New York, USA; Division of Infectious Diseases, Department of Internal Medicine, The University of Texas Health Science Center at Houston, McGovern Medical School, Houston, Texas, USA; Division of Infectious Diseases, Department of Medicine, University of Miami Miller School of Medicine, Miami, Florida, USA; Miami Clinical and Translational Science Institute, University of Miami Miller School of Medicine, Miami, Florida, USA; Division of Pulmonary and Critical Care Medicine, Department of Internal Medicine, The University of Texas Health Science Center at Tyler, UTHealth East Texas, Tyler, Texas, USA; Section of Infectious Diseases, Department of Internal Medicine, Yale University School of Medicine, New Haven, Connecticut, USA; Division of Infectious Diseases, Department of Medicine, Albert Einstein College of Medicine, Montefiore Medical Center, Bronx, New York, USA; Harold and Muriel Block Institute for Clinical and Translational Research, Albert Einstein College of Medicine and Montefiore Medical Center, Bronx, New York, USA; Leon H. Charney Division of Cardiology, Department of Medicine, NYU Grossman School of Medicine, New York, New York, USA; Division of Infectious Diseases, Department of Medicine, Albert Einstein College of Medicine, Montefiore Medical Center, Bronx, New York, USA; Department of Microbiology and Immunology, Albert Einstein College of Medicine, Bronx, New York, USA; Division of Infectious Diseases, Department of Medicine, NYU Grossman School of Medicine, New York, New York, USA; Department of Microbiology, NYU Grossman School of Medicine, New York, New York, USA

**Keywords:** acute hypoxic COVID-19, COVID-19 convalescent plasma, long COVID, PASC, Patient-Reported Outcomes Measurement Information System

## Abstract

**Background:**

The long-term effect of coronavirus disease 2019 (COVID-19) acute treatments on postacute sequelae of severe acute respiratory syndrome coronavirus 2 (SARS-CoV-2) infection (PASC) is unknown. The CONTAIN-Extend study explores the long-term impact of COVID-19 convalescent plasma (CCP) therapy on postacute sequelae of SARS-CoV-2 infection (PASC) symptoms and general health 18 months following hospitalization.

**Methods:**

The CONTAIN-Extend study examined 281 participants from the original CONTAIN COVID-19 trial (CONTAIN-RCT, NCT04364737) at 18 months post–hospitalization for acute COVID-19. Symptom surveys, global health assessments, and biospecimen collection were performed from November 2021 to October 2022. Multivariable logistic and linear regression estimated associations between the randomization arms and self-reported symptoms and Patient-Reported Outcomes Measurement Information System (PROMIS) scores and adjusted for covariables, including age, sex, race/ethnicity, disease severity, and CONTAIN enrollment quarter and sites.

**Results:**

There were no differences in symptoms or PROMIS scores between CCP and placebo (adjusted odds ratio [aOR] of general symptoms, 0.95; 95% CI, 0.54–1.67). However, females (aOR, 3.01; 95% CI, 1.73–5.34), those 45–64 years (aOR, 2.55; 95% CI, 1.14–6.23), and April–June 2020 enrollees (aOR, 2.39; 95% CI, 1.10–5.19) were more likely to report general symptoms and have poorer PROMIS physical health scores than their respective reference groups. Hispanic participants (difference, −3.05; 95% CI, −5.82 to −0.27) and Black participants (−4.48; 95% CI, −7.94 to −1.02) had poorer PROMIS physical health than White participants.

**Conclusions:**

CCP demonstrated no lasting effect on PASC symptoms or overall health in comparison to the placebo. This study underscores the significance of demographic factors, including sex, age, and timing of acute infection, in influencing symptom reporting 18 months after acute hypoxic COVID-19 hospitalization.

Despite the declaration ending the Federal Coronavirus Disease 2019 (COVID-19) Public Health Emergency in the United States on May 11, 2023 [1], a significant number of individuals who were infected with severe acute respiratory syndrome coronavirus 2 (SARS-CoV-2) experienced postacute sequelae of SARS-CoV-2 infection (PASC), also known as “long COVID.” In October 2021, the World Health Organization (WHO) introduced a consensus case definition for long COVID as the persistence or development of new symptoms 3 months after the initial SARS-CoV-2 infection that lasts for a minimum of 2 months and cannot be explained by any other cause [2]. The US Center for Clinical Diseases estimated that ∼6.9% of adults with SARS-CoV-2 infection ever had long COVID in 2022 [3]. While hypotheses range from persistent viral antigens to immune dysregulation, the risk factors, mechanisms, and treatments for PASC are poorly understood [4–6].

CONTAIN COVID-19 was a randomized controlled trial (CONTAIN-RCT) designed to investigate the efficacy and safety of COVID-19 convalescent plasma (CCP) compared with a placebo (normal saline) in hospitalized patients with acute hypoxic COVID-19 [7]. While overall the outcomes of CCP and placebo recipients did not differ, subgroup analyses demonstrated heterogeneity of treatment effect. Participants in the CCP group who were enrolled in the early stages of the pandemic or did not receive other COVID-19 therapies exhibited improved clinical outcomes. To investigate the relationship between receipt of CCP and the incidence of PASC symptoms among CONTAIN participants, the study reported herein, CONTAIN-Extend, was undertaken to assess PASC symptoms and general health in the CONTAIN-RCT cohort 18 months after randomization.

## METHODS

### Study Design and Setting

CONTAIN-RCT [7], conducted from April 2020 to March 2021, completed its final follow-up at 3 months postrandomization. A protocol amendment in August 2021 introduced CONTAIN-Extend, a prospective cohort study evaluating the effect of CCP vs placebo on COVID-19 symptoms and global health at 18 months (±3 months) following the initial CONTAIN randomization (Supplementary Data 1). The visits took place between November 2021 and October 2022. The study participants were enrolled from 16 of the 21 CONTAIN-RCT study sites that agreed to participate in CONTAIN-Extend located in Manhattan, Bronx, Brooklyn, and Long Island (New York), New Haven (Connecticut), Miami (Florida), and Houston and Tyler (Texas). The 18-month visit was approved by the institutional review boards (IRBs) of each participating site, with trial oversight provided by the New York University CONTAIN Coordinating Center.

### Study Population

Participants who were previously randomized in CONTAIN-RCT, had not withdrawn from the study, and had survived through 3-month follow-up, were eligible for the study and were approached by the study team for recruitment. By the time of the encounter, the study participants and investigators were unblinded regarding the randomization arm.

### Patient Consent and Study Procedures

Participants were recruited through phone calls, Electronic Health Record Patient Portal (EPIC's MyChart) messages and/or by IRB-approved letters. Verbal or written consent was obtained from the participants or legally authorized representatives if the participant lacked capacity. No special measures were taken to ensure balance of participant characteristics across the study arms. This was a minimal-risk study, and a waiver for documented written consent was granted by the IRB. Participants who were unreachable after multiple attempts were considered lost to follow-up.

Interviews with the participants were conducted either via phone or in person using a questionnaire developed by the CONTAIN investigators based on the literature at the time of protocol development in August 2021 (Supplementary Data 2) to assess reported COVID-19-related symptoms in the week before survey completion. Information on COVID-19 vaccination and adverse events between the 3-month and 18-month visits, including pneumonia, re-admission, and SARS-CoV-2 re-infection, was collected. Nasopharyngeal swabs and peripheral blood samples were obtained from consenting participants for SARS-CoV-2 polymerase chain reaction testing, assessment of inflammatory and hematological markers (ie, fibrinogen, lactate dehydrogenase, ferritin, C-reactive protein, and absolute lymphocyte count), and potential future research.

The Patient-Reported Outcomes Measurement Information System (PROMIS)–10 (version 1.2) in English and Spanish evaluated participants’ global physical and mental health [8] (Supplementary Data 2). Participants completed the survey in person on paper or via online Research Electronic Data Capture (REDCap). The PROMIS global physical score addresses physical health and functioning, pain, and fatigue, while the mental score assesses quality of life, mental health, social satisfaction, and emotional issues. Scores obtained from a 5-point Likert scale are translated to a T-score, with 50 as the US population mean and an SD of 10 [9].

Data on demographics, medical conditions, COVID-19 severity (as determined by the 11-point WHO Ordinal Scale for Clinical Improvement) [10], SARS-CoV-2 spike protein serostatus, and laboratory data at the time of randomization (ie, baseline) were extracted from the CONTAIN-RCT database. Race and ethnicity data were obtained from the medical records, as reported by the participants using predefined categories required by the funding agency, and were reported to describe racial and ethnic representation within the cohort, which may impact the generalizability of the findings.

### Outcomes

The outcomes assessed were self-reported COVID-related symptoms and global physical and mental health indices during the 18-month visit.

### Statistical Analysis

Continuous baseline covariables were reported using median and interquartile range (IQR), and the PROMIS outcome measures used mean and SD; categorical baseline covariables used frequency and percentage. Group differences were tested using the Mann-Whitney or χ^2^ test. Self-reported symptoms as a binary variable (yes/no) were categorized into 4 systems: general (fever or fatigue); respiratory (sore throat, rhinorrhea, nasal congestion, cough, dyspnea, or chest pain); gastrointestinal (diarrhea, nausea/vomiting, or abdominal pain); and neurological (myalgia, headache, loss of smell, or loss of taste).

We compared system-based symptoms and PROMIS scores at 18 months across randomization arms using multivariable logistic and linear regression models, respectively. These models adjusted for clinically relevant factors that were selected a priori. Covariables included demographics (age, sex, race/ethnicity), baseline WHO score, and CONTAIN enrollment quarter and sites. Results were presented as adjusted odds ratios (aORs) or mean differences with 95% CIs. Changes in inflammatory and hematological markers from baseline to 18 months were compared across randomization arms. The changes were then used as predictors of symptom outcome in a multivariable logistic regression model. All analyses were conducted using R (version 4.3.1).

## RESULTS

### CONTAIN-Extend Cohort

**Figure 1.**
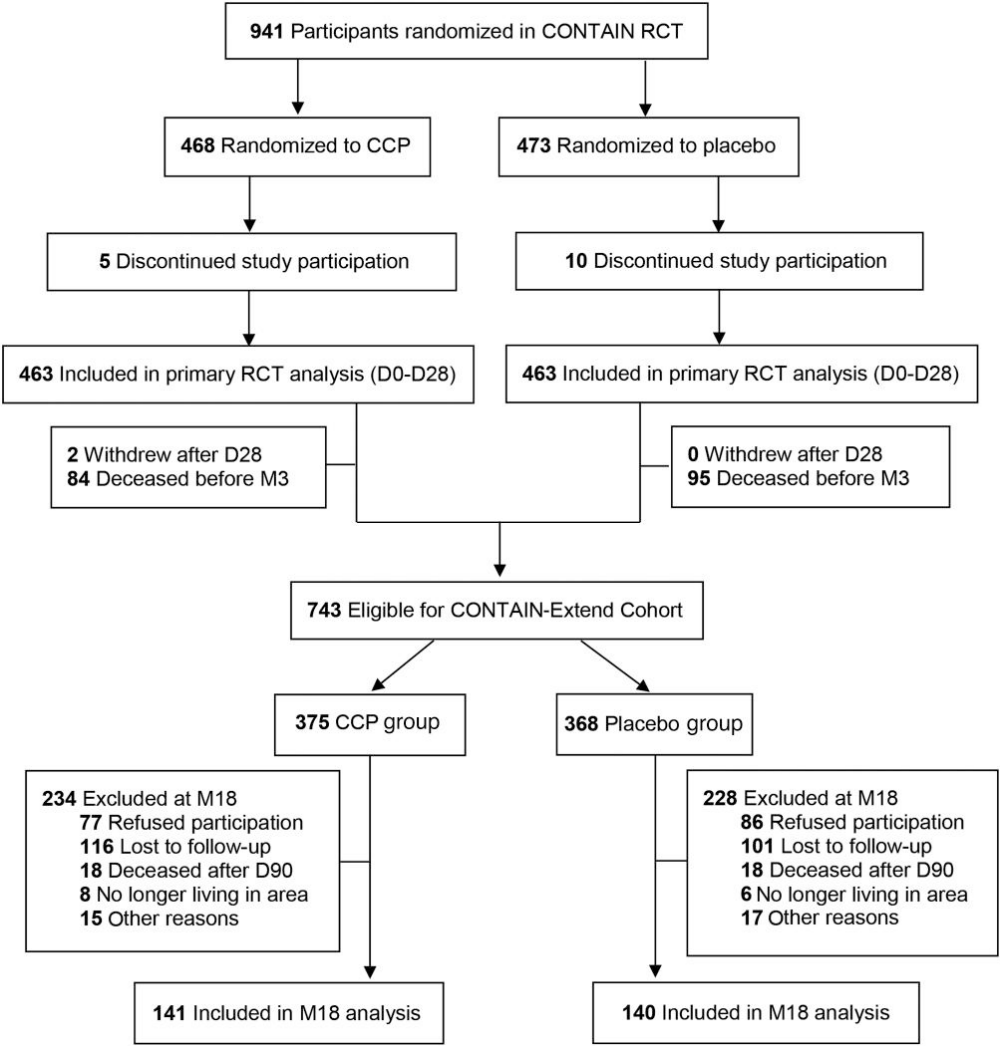
Patient enrollment and treatment assignment of the CONTAIN-Extend cohort. D28, M3, and M18 indicate 28 days, 3 months, and 18 months after randomization, respectively.

Abbreviations: CCP, coronavirus disease 2019 convalescent plasma; RCT, randomized controlled trial.

Between November 2021 and October 2022, 743 eligible participants, that is, those who survived until the final 3-month follow-up of CONTAIN and had not withdrawn, were contacted and invited to participate, among whom 281 (37.8%) provided consent and were enrolled, with 141 in the CCP arm and 140 in the placebo arm. A total of 163 (21.9%) declined to participate, 217 (29.2%) were lost to follow-up, and 36 (4.8%) died after the 3-month trial visit (Figure 1). Of all enrolled participants, 178 (63.3%) were recruited from New York City (NYC) sites (Supplementary Data 3; Supplementary Table 1).

Baseline demographics and clinical characteristics were generally well balanced between the CCP and placebo arms, except for a higher proportion of Asian participants (10.6% vs 1.4%) and individuals with a history of diabetes (34.8% vs 23.6%) in the CCP arm (Table 1). The study cohort participants who were eligible, alive, and not enrolled in the CONTAIN-Extend cohort were referred to as “nonparticipants.” Compared with nonparticipants, CONTAIN-Extend participants at baseline were younger (median age [IQR], 59 [50–67] vs 63 [50–73] years), had a higher proportion of females (44.5% vs 41.8%), and had a lower prevalence of comorbid conditions, including pulmonary disease (7.5% vs 11.9%) and diabetes (29.2% vs 37.0%) (Supplementary Table 2). Most CONTAIN-Extend participants (241, 85.8%) were enrolled in CONTAIN-RCT after the second quarter (June) of 2020, 254 (90.4%) had received at least 1 dose of COVID-19 vaccine, and 12 (4.3%) had SARS-CoV-2 re-infection, with 6 from each randomization arm. No adverse event differences were observed between the CCP and placebo groups (Supplementary Table 3).

**Table 1.** Demographic and Clinical Characteristics of the CONTAIN-Extend Cohort by Randomization Arm

	Placebo	CCP	*P* Value
No.	140	141	
Age, median (IQR), y	59 (50–68.3)	58 (49–67)	.55
Age, categorical, No. (%)			
<45 y	25 (17.9)	26 (18.4)	.96
45–65 y	69 (49.3)	71 (50.4)	
>65 y	46 (32.9)	44 (31.2)	
Sex, No. (%)			
Male	74 (52.9)	82 (58.2)	.44
Female	66 (47.1)	59 (41.8)	
Race/ethnicity,^a^ No. (%)			
Asian	2 (1.4)	15 (10.6)	.02
Hispanic	62 (44.3)	54 (38.3)	
Non-Hispanic Black	22 (15.7)	24 (17.0)	
Non-Hispanic White	45 (32.1)	43 (30.5)	
Other/unknown^b^	9 (6.4)	5 (3.5)	
BMI,^c^ median (IQR), kg/m^2^	31.1 (26.7–36.5)	31.6 (26.6–36.6)	.99
Enrollment quarter, No. (%)			
2020 Q2	18 (12.9)	22 (15.6)	.63
2020 Q3–2021 Q5	122 (87.1)	119 (84.4)	
WHO score at randomization, No. (%)			
5	111 (79.3)	109 (77.3)	.80
6	29 (20.7)	32 (22.7)	
Baseline spike IgG serostatus,^d^ No. (%)			
Negative	34 (24.3)	41 (29.1)	.09
Positive	85 (60.7)	68 (48.2)	
N/A	21 (15.0)	32 (22.7)	
Comorbidities, No. (%)			
Pulmonary	10 (7.1)	11 (7.8)	1.00
Hypertension	77 (55.0)	81 (57.4)	.77
Cardiovascular	50 (35.7)	50 (35.5)	1.00
Diabetes	33 (23.6)	49 (34.8)	.05
Chronic kidney disease	9 (6.4)	18 (12.8)	.11
Concurrent medications, No. (%)			
Remdesivir	86 (61.4)	85 (60.3)	.94
Corticosteroids	114 (81.4)	118 (83.7)	.73
Therapeutic anticoagulation	113 (80.7)	115 (81.6)	.98
Months between randomization and 18-mo visit, median (IQR)	17.0 (15.7–19.9)	17.6 (15.8–19.8)	.67
COVID-19-vaccinated individuals,^e^ No. (%)	129 (92.1)	125 (88.7)	.43
COVID-19 re-infection, No. (%)	6 (4.3)	6 (4.3)	>.99

*P* value represents a comparison between the placebo and CCP groups.

Abbreviations: BMI, body mass index; CCP, COVID-19 convalescent plasma; COVID-19, coronavirus disease 2019; FDA, Food and Drug Administration; IgG, immunoglobulin G; IQR, interquartile range; N/A, not available; Q, quarter; SARS-CoV-2, severe acute respiratory syndrome coronavirus 2; WHO, World Health Organization.

^a^Information on race and ethnic group was obtained from entries in the medical record, as reported by the patients.

^b^Other included mixed race, American Indian or Alaska Native, and Native Hawaiian or other Pacific Islander.

^c^BMI is calculated as weight in kilograms divided by height in meters squared.

^d^Defined as SARS-CoV-2 IgG titer >1:100 using in-house full-length spike protein enzyme-linked immunosorbent assay.

^e^Includes all COVID-19 vaccines authorized or approved by the FDA: Pfizer-BioNTech, Moderna, and Johnson & Johnson's Janssen.

### Symptom Assessment

Analysis of the 18-month visit of the CONTAIN-Extend cohort showed that 156 (55.5%) participants reported at least 1 predefined symptom. The highest proportion of symptoms reported were respiratory (37.7%), followed by general (28.8%), neurological (22.1%), and gastrointestinal (16.4%) symptoms (Supplementary Table 4).

### Factors Associated With Symptoms

**Figure 2.**
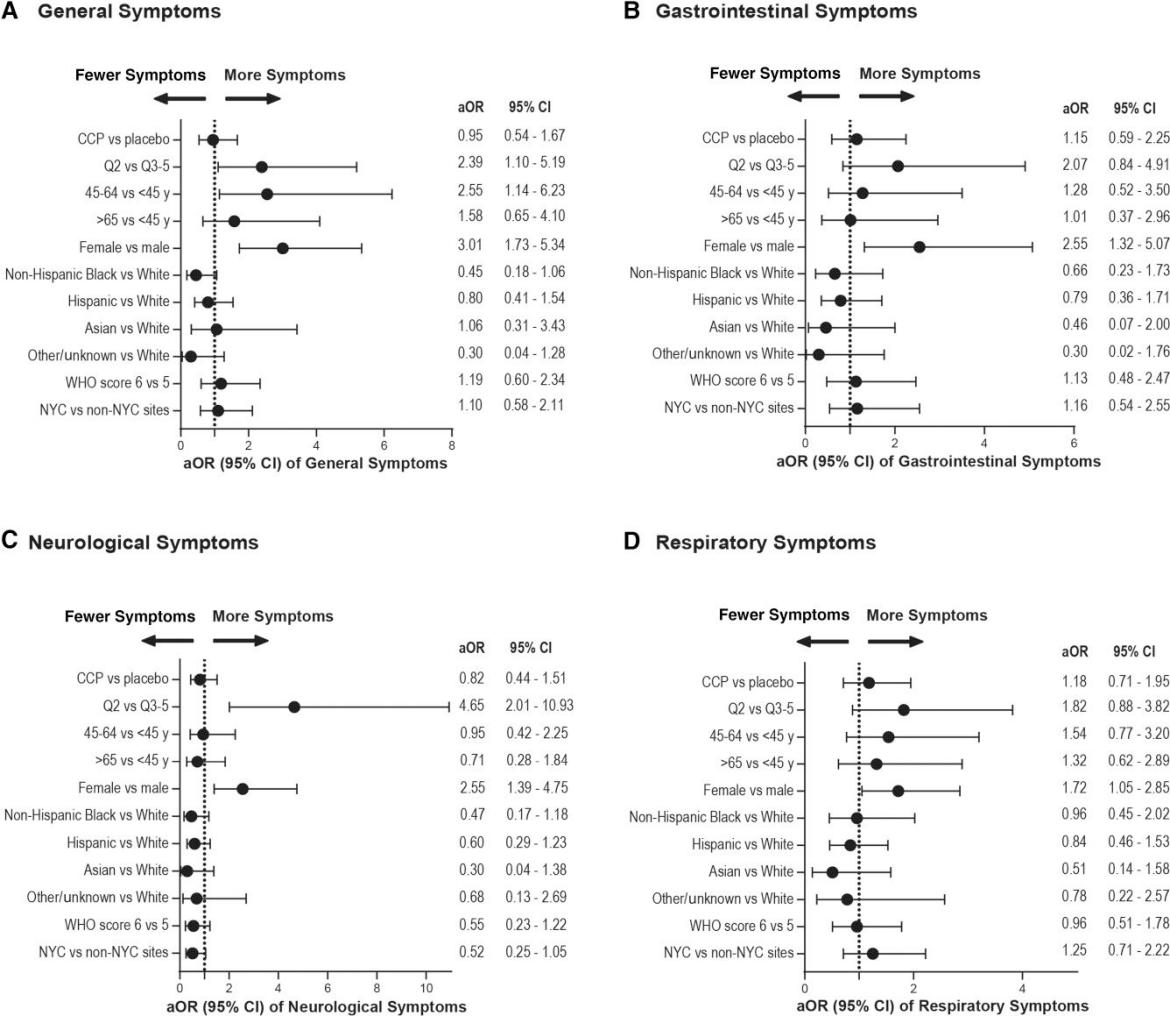
Forest plots demonstrating the association of clinical variables with 18-month symptoms: (A) general, (B) gastrointestinal, (C) neurological, and (D) respiratory. Adjusted using a multivariable logistic regression model. Figure axis labels indicate the primary category being tested against the reference category.

Abbreviations: aOR, adjusted odds ratio; CCP, coronavirus disease 2019 convalescent plasma; WHO, World Health Organization.

In the multivariable logistic regression analysis, no association was observed between the randomization arm and all 4 symptom groups, adjusted for age, race/ethnicity, enrollment quarter, baseline WHO score, and site (general: aOR, 0.95; 95% CI, 0.54–1.67; gastrointestinal: aOR, 1.15; 95% CI, 0.59–2.25; neurological: aOR, 0.82; 95% CI, 0.44–1.51; respiratory: aOR, 1.18; 95% CI, 0.71–1.95). Notably, females were more likely than males to have symptoms at 18 months, including general (aOR, 3.01; 95% CI, 1.73–5.34), gastrointestinal (aOR, 2.55; 95% CI, 1.32–5.07), neurological (aOR, 2.55; 95% CI, 1.39–4.75), and respiratory (aOR, 1.72; 95% CI, 1.05–2.85) symptoms. Individuals enrolled in the second quarter of 2020 (Q2) had a higher occurrence of general (aOR, 2.39; 95% CI, 1.10–5.19) and neurological (aOR, 4.65; 95% CI, 2.01–10.93) symptoms at 18 months compared with those enrolled in quarters Q3–Q5. In addition, compared with participants aged <45, those aged 45–64 years were more likely to have general symptoms (aOR, 2.55; 95% CI, 1.14–6.23) (Figure 2). There were no significant differences in symptoms based on race/ethnicity, baseline WHO score, baseline spike-antibody serostatus, vaccination status, or site of enrollment.

### Global Physical and Mental Health Outcomes

Among CONTAIN-Extend participants, 277 (98.6%) completed the PROMIS survey. The average physical T score (SD) was 46.4 (9.9), below the US average, whereas the mental T score matched the national mean (SD) at 50 (8.8). Lower physical scores were observed in females (mean [SD], 44.1 [10.4]), Hispanic participants (mean [SD], 44.9 [10.0]), Black participants (mean [SD], 44.4 [9.8]), and those enrolled during Q2 (April–June 2020: mean [SD], 43.4 [10.4]) compared, respectively, with males, White participants, and those enrolled in Q3–Q5 (Supplementary Tables 5 and 6).

**Figure 3.**
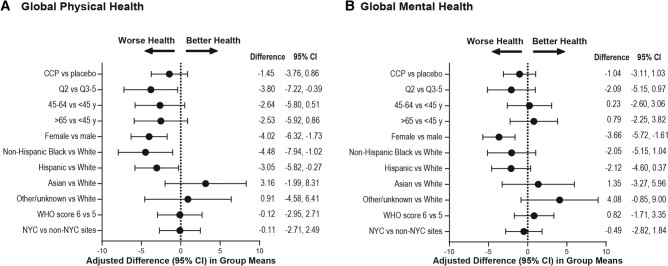
Forest plots demonstrating the association between clinical variables and PROMIS-10 T scores: (A) global physical health and (B) global mental health. Adjusted using a multivariable linear regression model. Figure axis labels indicate the primary category being tested against the reference category.

Abbreviations: aOR, adjusted odds ratio; CCP, coronavirus disease 2019 convalescent plasma; WHO, World Health Organization.

After adjusting for participant-level characteristics, there was no apparent difference in mean global physical (difference, −1.45; 95% CI, −3.76 to 0.86) or mental health scores (difference, −1.04; 95% CI, −3.11 to 1.03) across the randomization arms. Females reported lower physical health than males (difference, −4.0; 95% CI, −6.3 to −1.7). Hispanic participants (difference, −3.05; 95% CI, −5.82 to −0.27) and Black participants (difference, −4.48; 95% CI, −7.94 to −1.02) reported lower physical health than White participants. Finally, participants enrolling in Q2 had lower physical health scores than those enrolling in Q3–Q5 (difference, −3.80; 95% CI, −7.22 to −0.39). Statistical differences were not observed for global mental health scores, except for females compared with males (difference, −3.66; 95% CI, −5.72 to −1.61) (Figure 3).

Model estimates indicate significant alignment between all CONTAIN-Extend symptom group measures and both physical and mental PROMIS scales (Supplementary Figure 1).

### Inflammatory and Hematological Markers

At the 18-month visit, levels of fibrinogen, lactate dehydrogenase, ferritin, and C-reactive protein were lower, while lymphocyte counts were higher than at enrollment in CONTAIN-RCT, regardless of the randomization arm (Supplementary Table 7). In a multivariable model adjusted for covariables, the changes in the markers from baseline to month 18 generally showed no significant association with symptoms (data not shown).

## DISCUSSION

In this prospective multicenter observational study of 281 individuals who had previously participated in CONTAIN-RCT [7], there were no discernable differences in the occurrence of COVID-19-related symptoms or physical or mental PROMIS scores at 18 months following hospitalization between CCP and placebo recipients. A considerable proportion (56%) of participants experienced symptoms, predominantly respiratory, with more physical impairment and lesser mental impact at 18 months, regardless of their randomization arm. Notably, female participants, those aged 45–64 years, and individuals enrolled during Q2 (April–June 2020) were more likely to report general symptoms at 18 months compared with their respective comparison groups, a finding that was also consistent with PROMIS outcomes. The PROMIS score additionally revealed greater physical impairment among Hispanic and Black participants than White participants.

The absence of an overall difference in PASC outcomes between CCP and placebo in the CONTAIN-Extend cohort may be attributed to enrollment of hospitalized patients who were hypoxic and required oxygen supplementation (WHO 5 and WHO 6) at the time of randomization and CCP transfusion, making them unlikely to benefit from the potential antiviral activity of CCP [11–13]. Furthermore, while CONTAIN-RCT CCP recipients might have benefited during April–June 2020 (Q2), which was retrospectively determined to have been the time period with high CCP neutralizing titer [7], most CONTAIN Q2 participants were not enrolled in CONTAIN-Extend. Hence, we are not able to assess the effect of high-titer CCP on PASC symptoms.

Viral persistence or a reservoir of active virus has been implicated as a driver of PASC symptoms [6, 14]. In a randomized controlled trial (RCT) that examined CCP efficacy in outpatients, there was a trend toward fewer PASC symptoms in participants treated with CCP within 5 days of symptom onset compared with those treated later [15]. Treatment with nirmatrelvir [16], remdesivir [17], and metformin [18] has also been associated with reduced PASC symptoms, although remdesivir did not associate with symptom reduction during a 1-year follow-up of an RCT involving hospitalized COVID-19 patients [19]. While low production of SARS-CoV-2 antibodies and a minimal immune response during acute disease were associated with longer post-COVID-19 symptom duration [20, 21], we did not find an association between baseline SARS-CoV-2 spike-antibody serostatus and symptoms at 18 months, with the caveat that this analysis was limited by sample availability, particularly from Q2, where a benefit of CCP was observed in the original CONTAIN-RCT cohort [7]. Apart from antiviral effects, CCP influenced the evolution of endogenous SARS-CoV-2 antibody responses by modulating inflammatory spike protein and enhancing nucleocapsid antibody responses [22]. This could have implications for PASC symptoms if they are in part due to chronic inflammation, which will be investigated in a future study. Although some have reported inflammatory cytokines as possible drivers of PASC [23, 24], we did not find a meaningful association between the prespecified inflammatory markers and symptoms.

Consistent with previous studies, we found a significant association between female sex and symptoms, as well as with global physical and mental health at 18 months. This has been attributed to biological, immunological, socioeconomic, and psychological factors that our study was not designed to assess [25–28]. Interestingly, studies indicate that females exhibit a stronger acute-phase antibody response [29], while males maintain higher antibody levels over time post-COVID-19 [30] but also experience a more pronounced decline after vaccination [31]. The role of antibodies in explaining PASC symptoms through immune dysregulation remains speculative [32].

Notably, our study indicates that Hispanic and Black participants might have experienced a more significant impact of COVID-19 on their global physical health compared with White participants. This disparity could stem from these groups disproportionately being impacted earlier in the pandemic with barriers to timely health care access [33], making them more vulnerable to long-term health consequences [34]. Interestingly, this racial and ethnic difference was not reflected in CONTAIN-Extend symptom assessments. While racial and ethnic differences in global physical impairment might be related to chronic conditions, COVID-related symptoms may have been under-reported, especially in light of a large NYC cohort study that highlighted racial and ethnic differences in symptom reporting [34].

### Strengths and Limitations

The strengths of our study include its prospective design, the use of a prespecified questionnaire, and a longer follow-up duration compared with other studies conducted thus far. We were able to recruit a significant proportion of eligible participants. Additionally, a high proportion of Hispanic and Black participants completed the bilingual PROMIS survey. These groups are disproportionally impacted by the disease [33], yet often under-represented in standardized surveys [35]. Overall, findings from our symptom survey correlated well with the PROMIS global physical health scores in the adjusted model, supporting the reliability of our symptom assessment using an externally validated instrument [36].

Our study has important limitations. Being an observational survey–based study reliant on self-reported symptoms, our results may have been affected by biases, including recall bias, response bias, and measurement bias, which may correlate with time lapse between initial enrollment and follow-up. For example, the weaker correlation between COVID-related symptoms and PROMIS mental health scores, despite reports of long-term neuropsychiatric consequences [37], might stem from latent factors such as stigma [38] or response bias or may reflect a more transient nature of psychiatric symptoms after acute COVID-19 [39]. The limitation of the PROMIS-10 tool, indicated by its weak correlation with Quality of Life in Neurological Disorders (NeuroQoL) measures [40], particularly in assessing cognitive impairment noted in other PASC studies [41], might also be a contributing factor.

We assessed symptoms in participants who consented to both the original RCT and its extension, with 51% declining or lost to follow-up, potentially selecting those with health-seeking behaviors [42], as suggested by the 90% voluntary vaccination rate. Unaccounted factors related to social determinants, such as compensation, staff diversity, and language translation ease, beyond the scope of our analysis, may also have influenced the decision to participate and our outcome measures. Survivorship bias is possible, as individuals who received CCP compared with placebo may have had a mortality benefit, or specific groups, such as females and middle-aged individuals, may have had higher survival rates during acute disease (Supplementary Table 2), at the expense of more complicated hospital courses and subsequent prolonged symptoms.

The extended 18-month time gap raises the risk of unaccounted or latent variables. While we observed a modest 4% reinfection rate compared with other studies [43, 44], various regimens and numbers of doses of SARS-CoV-2 vaccination might have influenced the outcome, given that the available evidence suggests that vaccination may mitigate PASC symptoms [45–47]. Furthermore, the time difference between acute infection and vaccination, which narrowed over the period of CONTAIN-Extend, might affect symptom reports, especially if vaccination influenced symptom manifestation. In fact, individuals enrolled early in the pandemic, with a longer gap to vaccination, were more likely to report symptoms, notably neurological symptoms, and have global physical health impairment. Neurological symptoms have been recognized as potential delayed complications of COVID-19 [48, 49].

We designed the study before the WHO's case definition of PASC was put forth in October 2021, which includes the criterion of symptom persistence for at least 8 weeks [2]. Similarly, most PROMIS items were based on a 7-day recall period [36]. Therefore, we cannot infer WHO-defined PASC based on the symptoms evaluated in our study, as the assessment did not consider the minimum duration of 8 weeks. Furthermore, as the definition of PASC continues to develop beyond symptom categorizations [50] and explores pathogenesis and diverse mechanisms [5, 51], revisiting our cohort using future PASC definitions could potentially reveal new insights. Most critically, we did not have a control group consisting of individuals who did not have COVID-19 but experienced serious illnesses requiring hospitalization that can lead to long-term complications [52]. Furthermore, the PROMIS survey assesses general physical and mental health parameters and is not specifically tailored to COVID-19.

## CONCLUSIONS

Our findings demonstrate that a significant proportion of COVID-19 survivors infected with SARS-CoV-2 before widespread use of vaccination experience a range of long-term symptoms. While CCP, compared with placebo, may have shown clinical benefits in the CONTAIN-RCT cohort early in the pandemic, it did not affect the incidence of long-term symptoms at 18 months. However, participants hospitalized for hypoxic COVID-19 early in the pandemic were more likely to report neurological symptoms at month 18, regardless of their randomization arm, compared with those hospitalized later. Mechanistic insight into the factors, especially sex, that govern the development of PASC symptoms is needed.

## Supplementary Material

ofad686_Supplementary_Data

## Supplementary Data

Supplementary materials are available online. Supplementary materials consist of data provided by the author that are published to benefit the reader. The posted materials are not copyedited. The contents of all supplementary data are the sole responsibility of the authors. Questions or messages regarding errors should be addressed to the author.
