# Supplementary material for: COVID-19 Convalescent Plasma Therapy: Long-term Implications
Source: Open Forum Infect Dis. 2023 Dec 29;11(1):ofad686. doi: 10.1093/ofid/ofad686 (PMC10807994; doi:10.1093/ofid/ofad686)
Supplement: ofad686_Supplementary_Data [file ofad686_supplementary_data.pdf]

Supplements

		Page
**Supplement 1:**		
	CONTAIN-Extend Study Protocol (Addendum to CONTAIN COVID-19 Protocol v3.2)	2
**Supplement 2:**		
	The CONTAIN-Extend Symptom Questionnaire	6
	PROMIS® Scale v1.2 – Global Health	8
	PROMIS®–29 Profile v2.1	10
**Supplement 3:**		
	**Figure S1.** Forest Plots for the Association of System-Based Symptoms with PROMIS-10 T Scores: (A) Global Physical Health and (B) Global Mental Health, Multivariable Linear Regression Adjusted for Covariables.	14
	**Table S1.** Demographic and Clinical Characteristics of the CONTAIN-Extend Cohort by Enrollment Site	15
	**Table S2.** Demographic and Clinical Characteristics of the CONTAIN-Extend Cohort vs. Non-Participants	17
	**Table S3.** Adverse Events in the CONTAIN-Extend Cohort	18
	**Table S4.** Symptoms in the CONTAIN-Extend Cohort by Randomization Arm at Baseline and Month 18	19
	**Table S5.** Global Physical and Mental Health T-Scores from PROMIS Survey, breakdown by Clinical Variables	20
	**Table S6.** Mean Scores of the PROMIS Items for Global Physical and Mental Health Assessment	21
	**Table S7.** Inflammatory Markers in the CONTAIN-Extend Cohort at Baseline and Month 18	22
**Supplement 4:**		
	Data Sharing Statement	23
**Supplement 5:**		
	The CONTAIN-Extend Study Group	24

Supplement 1

Convalescent Plasma to Limit Coronavirus Associated Complications: A Randomized Blinded Phase 2 Study Comparing the Efficacy and Safety of Anti-SARS-CoV-2 Plasma to Placebo in Covid-19 Hospitalized Patients (CONTAIN COVID-19) Addendum:

Extension of CONTAIN COVID-19 to include an additional follow-up visit

Addendum to Protocol Version: 3.2

Protocol Date: April 4, 2021

Addendum Date: November 12, 2021

IND 20427

ClinicalTrials.gov Identifier: NCT 04364737

PROTOCOL SUMMARY

Addendum Overview:
An additional follow-up visit will be conducted around 18 months with a grace period between 15 to 21 months after the study participant received study product, either COVID-19 convalescent plasma (CCP) or placebo (saline solution). The additional visit will be an extension of the CONTAIN protocol and will be referred as CONTAIN-Extend.

Introduction and Rationale: Patients who recover from COVID-19 may continue to experience symptoms after initial recovery [1]. The phenomenon of post-acute COVID syndrome (PACS) in patients sometimes called 'long haulers' has raised important questions about the pathogenesis of persistent symptoms and the immunological factors that might drive them [2]. The effect of COVID-19 therapies, including CCP on subsequent development of PACS has not been examined. The results of CONTAIN showed that CCP may have had an effect early in the pandemic before corticosteroids and Remdesivir became standard of care for many hospitalized COVID-19 patients. There was no correlation between CCP SARS-CoV-2 antibody titer or neutralization and clinical outcome at days 14 and 28 post-infusion. However, the effect of CCP may become evident later. For example, since we now know that COVID-19 can be a prolonged illness, later times of evaluation may provide a better understanding of whether patients experience true recovery and/or if CCP or other medications affect the development of chronic or persistent symptoms of COVID-19. Thus, we propose to extend the period of follow up of patients enrolled in CONTAIN to 15 to 21 months. This will allow the evaluation of long-term safety and efficacy of CCP and assessment of the longitudinal immune response to SARS-CoV-2 infection via various exploratory projects, which include an assessment of the serology, B and T cell phenotyping, and single cell RNA sequencing. The goal is to characterize the immunological profile of recovered patients by treatment arm, CCP versus placebo. We recognize that participation in this extended study is voluntary, so the balance of patient characteristics generated by the initial randomization may be broken; before conducting our analyses, we will assess whether this is the case.

Study Population: All study participants who were randomized, survived, and did not withdraw consent in CONTAIN will be eligible to participate in CONTAIN-Extend. Subjects who completed the first part of the CONTAIN study will be re-contacted by study staff to review consent of extension visit. The subjects/LARs who are re-consented via phone will consent verbally and study staff will document consent in subject chart. Participants who do not answer initial call attempt will be sent an IRB-approved MyChart message to alert them of additional follow-up visit and contact information for completion of the phone consent. Due to limited availability of clinic space, subjects will be consented and complete the survey via phone call, and will go to outpatient labs for blood collection and nasal swab. The verbal re-consent of subjects is being obtained under a Waiver of Documentation of Informed Consent. Participants who have died after their Day 90 Follow-Up Visit but prior to re-consent of Contain-Extend may have their death information collected if readily available via EMR, family correspondence, or public record. This information includes date and cause of death, if available.

Study Composition: NYU Langone Heath will serve as the Clinical and Data Coordinating Center for the study under IND 20427.

Data Collection and Study Procedures: The following will be assessed:

I. Laboratory and Imaging Data via outpatient laboratory (Collected, all or some, as feasible by individual study sites from patients that consent to providing specimens)
   1. Laboratory Data:
      - Hematologic markers: CBC with differential (neutrophil, lymphocyte counts and platelet count explicitly recorded), LDH, D-dimer, fibrinogen, ferritin, Lymphocyte subsets
      - Metabolic markers: Complete metabolic panel, LFT (optional; can record most recent available value (between 15-21 months after randomization)
      - Inflammatory markers: CRP, cytokine panel (if possible)
      - SARS-CoV-2 PCR from nasopharyngeal swab
      - Quantitative serum immunoglobulins (IgG, IgM, IgA)
      - Whole blood: for plasma and PBMC isolation (Collected, as feasible by individual study sites from patients that agree to submit a specimen)
   2. Imaging: CT or Chest X-ray (optional to include if imaging is available in EMR and was obtained for other purposes).

II. Clinical Assessments via phone call, chart review, or in person if feasible.

1. Demographics: Updates to current demographics, which include race/ethnicity, BMI, comorbidities.
2. Disposition: home, hospital, nursing or other long-term facility, other.
3. COVID-19 symptom screen (fevers, cough, shortness of breath, etc., as per CONTAIN and PASC questionnaires)
4. Patient-Reported Outcomes Measurement Information System (PROMIS) 10 and 29 Questionnaires to assess quality of life, physical, and mental health.
5. Assessment of clinical status (WHO 11-point ordinal scale for clinical status)
6. Oxygen requirements: none vs other
7. Adverse events (including but not limited to mortality, hospitalizations, re-infections)
8. Concomitant medications
9. COVID-19 vaccination status

EXPLORATORY OBJECTIVES (addendum)

Exploratory objectives described in CONTAIN are unchanged, except for the following additional type of genetic study:

Single cell RNA sequencing (scRNAseq) of immune cells

PBMCs will be isolated from fresh blood obtained from participants, cryopreserved, and stored at each site's biorepository. Once all data is collected for enrolled patients, preserved cells will be subjected to flow cytometry for B and T cell phenotyping via cell surface markers, scRNAseq, and potentially other immune assays to assist in the immunoprofiling of patients' B and T cells. The aim is to understand if CCP affects patients' endogenous responses to SARS-CoV-2.

STUDY MODIFICATION (addendum)

Future Use of de-Identified Patient Information and Stored Specimens

Patients will be consented for the use of data and biological samples for current and future studies as previously described in Stage 1 of the protocol.

A subset of the immune cells isolated (PBMCs) in this visit will be subjected to single cell RNA sequencing in order to learn about how CCP affected the way in which patients produce antibodies or program their immune cells to respond to infection. No more than 550 mL of blood (equivalent amount defined as minimal risk) will be collected at outpatient laboratory. Collected serum, PBMCs, and nasopharyngeal swabs from patients that provided samples will be stored locally and shared with CONTAIN investigators who are performing downstream exploratory studies. De-identified patient samples and data will be shared between institutions and other collaborating centers for analysis in accordance to exploratory aims.

The collected serum, PBMCs, and nasopharyngeal swabs collected from NYU Langone Health patients, as well as the nasopharyngeal swabs from Albert Einstein School of Medicine patients, will be stored at the NYU Center for Biospecimen Research & Development (CBRD). The collected serum and PBMCs collected from Albert Einstein School of Medicine patients will be stored the Albert Einstein School of Medicine Molecular Cytogenetics Core.

Incidental Findings

The medical review and phlebotomy results for this research follow-up visit will be reviewed by the Research Team for Clinical Significance. Incidental findings discovered during this follow-up visit that are considered likely to be Clinically Significant or Medically Actionable by the Principal Investigator will be flagged and tracked in a tracker that the study team keeps in a secure shared drive. Participants will have the option to opt- in to be alerted of Incidental Findings in the Informed Consent Form, and this choice will be documented in the CRF database for tracking purposes. Participants who choose to be alerted of Incidental Findings will be contacted via phone call by a trained study team member (MD, DO, or RN), who will alert the participant of the finding and advise them to follow up with their Primary Care Provider (PCP). If participant cannot be contacted via phone after 3 attempts, a MyChart message will be sent to the participant and their PCP. If there are any life-threatening abnormalities that require immediate medical attention, a physician from the study team (MD or DO) will be assigned to call the participant. Participants who want to be alerted of incidental findings will also have their findings documented in their medical chart.

Compensation

For NYU participants: Participants will receive compensation in the form of three 100 dollar gift cards for the completion of each milestone of the follow-up visit, separated as questionnaire completion, blood collection, and nasopharyngeal swab collection. Participants will be compensated one gift card per milestone completion, for a maximum of 300 dollars.

Participant will be given the option to receive compensation as a Target gift card, Amazon gift card, or Bank of America gift card.

Both Target and Amazon gift cards may be sent to the participant via email to redeem online, and instructions will be sent to participant. The Bank of America gift card will be mailed to the participant's residence, along with the Bank of America Gift Card Policy to provide further information.

Unscheduled Visit

In the event that a lab collection is missed or incomplete, the participant may be asked to return to the site if the missed or incomplete lab collection is deemed necessary for Contain-Extend and it's exploratory objectives.
The amount of blood collection will remain the same and no additional risk will be introduced. To compensate the participant for their time and travel to site, 100 dollars in the form of a check will be sent to the participant (justified as 50 dollars for travel and parking and 50 dollars for compensation for time).

References:

1. Nasserie T, Hittle M, Goodman SN. Assessment of the Frequency and Variety of Persistent Symptoms Among Patients With COVID-19: A Systematic Review. *JAMA Network Open*. 2021;4(5):e2111417-e2111417. doi:10.1001/jamanetworkopen.2021.11417
2. Nalbandian A, Sehgal K, Gupta A, et al. Post-acute COVID-19 syndrome. *Nature Medicine*. 2021/04/01 2021;27(4):601-615. doi:10.1038/s41591-021-01283-z

Supplement 2

The CONTAIN-Extend Symptom Questionnaire

**In the past week, have you had any of these symptoms that are new or worsening compared to before you had COVID?**<br>**(May select more than one)**	**General or Constitutional Symptoms**<br>☐ Fever or chills<br>☐ Fatigue<br>☐ Difficulty sleeping<br>☐ Sleepiness during the day<br>☐ Feeling exhausted after walking<br><br>**HEENT**<br>☐ Sore throat<br>☐ Problems with swallowing or chewing<br>☐ Rhinorrhea<br>☐ Nasal congestion<br>☐ Problems with teeth/gums<br>☐ Change in hearing/ringing in the ears<br>☐ Hair loss<br>☐ Blurry vision<br><br>**Pulmonary**<br>☐ Cough<br>☐ Dyspnea/Shortness of Breath<br>☐ Trouble breathing<br>☐ Pain when breathing<br><br>**Cardiac**<br>☐ Chest Pain<br>☐ Palpitations/tachycardia<br><br>**Gastrointestinal**<br>☐ Poor appetite<br>☐ Nausea<br>☐ Vomiting<br>☐ Abdominal pain or cramps<br>☐ Diarrhea<br>☐ Constipation<br><br>**Genito/urological**<br>☐ Problems with urination<br>☐ Change in menstrual cycle<br><br>**Dermatological**<br>☐ Skin rash<br><br>**Neurological**<br>☐ Headache

	☐ Body aches, pains, or weakness<br>☐ Myalgia<br>☐ Loss of sense of smell<br>☐ Loss of sense of taste<br>☐ Feeling tingling or ‘pins and needles’ in hands and feet<br>☐ Dizziness<br>☐ Light headedness<br>☐ Fainting<br>☐ Feeling unsteady or off balance<br>☐ Seizures/fits<br>☐ Problems with concentration or attention<br>☐ Problems with memory<br>☐ Problems with speech<br><br>**Psychiatric**<br>☐ Anxiety or feeling on edge<br>☐ Shakiness/tremors<br>☐ Depression<br><br>**Other: Specify**

PROMIS® Scale v1.2 – Global Health

Global Health

Please respond to each question or statement by marking one box per row.

		Excellent	Very good	Good	Fair	Poor
Global01	In general, would you say your health is:	☐ 5	☐ 4	☐ 3	☐ 2	☐ 1
Global02	In general, would you say your quality of life is:...	☐ 5	☐ 4	☐ 3	☐ 2	☐ 1
Global03	In general, how would you rate your physical health?	☐ 5	☐ 4	☐ 3	☐ 2	☐ 1
Global04	In general, how would you rate your mental health, including your mood and your ability to think?	☐ 5	☐ 4	☐ 3	☐ 2	☐ 1
Global05	In general, how would you rate your satisfaction with your social activities and relationships?	☐ 5	☐ 4	☐ 3	☐ 2	☐ 1
Global09r	In general, please rate how well you carry out your usual social activities and roles. (This includes activities at home, at work and in your community, and responsibilities as a parent, child, spouse, employee, friend, etc.)......	☐ 5	☐ 4	☐ 3	☐ 2	☐ 1
		**Completely**	**Mostly**	**Moderately**	**A little**	**Not at all**
Global06	To what extent are you able to carry out your everyday physical activities such as walking, climbing stairs, carrying groceries, or moving a chair?	☐ 5	☐ 4	☐ 3	☐ 2	☐ 1

PROMIS® Scale v1.2 – Global Health

In the past 7 days…

		Never	Rarely	Sometimes	Often	Always
Global 10r	How often have you been bothered by emotional problems such as feeling anxious, depressed or irritable?	☐ 5	☐ 4	☐ 3	☐ 2	☐ 1

		None	Mild	Moderate	Severe	Very severe
Global 08r	How would you rate your fatigue on average?	☐ 5	☐ 4	☐ 3	☐ 2	☐ 1

Global07r	How would you rate your pain on average?	☐ 0 **No pain**	☐ 1	☐ 2	☐ 3	☐ 4	☐ 5	☐ 6	☐ 7	☐ 8	☐ 9	☐ 10 **Worst pain Imaginable**

PROMIS®–29 Profile v2.1

Please respond to each question or statement by marking one box per row.

	**Physical Function**	**Without any difficulty**	**With a little difficulty**	**With some difficulty**	**With much difficulty**	**Unable to do**
PFA11	Are you able to do chores such as vacuuming or yard work?.........................	☐ 5	☐ 4	☐ 3	☐ 2	☐ 1
PFA21	Are you able to go up and down stairs at a normal pace?.....................................	☐ 5	☐ 4	☐ 3	☐ 2	☐ 1
PFA23	Are you able to go for a walk of at least 15 minutes?...	☐ 5	☐ 4	☐ 3	☐ 2	☐ 1
PFA53	Are you able to run errands and shop?	☐ 5	☐ 4	☐ 3	☐ 2	☐ 1
	**Anxiety** **In the past 7 days…**	**Never**	**Rarely**	**Sometimes**	**Often**	**Always**
EDANX01	I felt fearful..	☐ 1	☐ 2	☐ 3	☐ 4	☐ 5
EDANX40	I found it hard to focus on anything other than my anxiety..............................	☐ 1	☐ 2	☐ 3	☐ 4	☐ 5
EDANX41	My worries overwhelmed me..................	☐ 1	☐ 2	☐ 3	☐ 4	☐ 5
EDANX53	I felt uneasy	☐ 1	☐ 2	☐ 3	☐ 4	☐ 5
	**Depression** **In the past 7 days...**	**Never**	**Rarely**	**Sometimes**	**Often**	**Always**
EDDEP04	I felt worthless	☐ 1	☐ 2	☐ 3	☐ 4	☐ 5
EDDEP06	I felt helpless..	☐ 1	☐ 2	☐ 3	☐ 4	☐ 5
EDDEP29	I felt depressed..	☐ 1	☐ 2	☐ 3	☐ 4	☐ 5
EDDEP41	I felt hopeless..	☐ 1	☐ 2	☐ 3	☐ 4	☐ 5
	**Fatigue** **During the past 7 days…**	**Not at all**	**A little bit**	**Somewhat**	**Quite a bit**	**Very much**
HI7	I feel fatigued..	☐ 1	☐ 2	☐ 3	☐ 4	☐ 5
AN3	I have trouble starting things because I am tired..	☐ 1	☐ 2	☐ 3	☐ 4	☐ 5

PROMIS®–29 Profile v2.1

	Fatigue<br>In the past 7 days…	Not at all	A little bit	Somewhat	Quite a bit	Very much
FATEXP41	How run-down did you feel on average?	☐ 1	☐ 2	☐ 3	☐ 4	☐ 5
FATEXP40	How fatigued were you on average?	☐ 1	☐ 2	☐ 3	☐ 4	☐ 5
	**Sleep Disturbance In the past 7 days…**	**Very poor**	**Poor**	**Fair**	**Good**	**Very good**
Sleep109	My sleep quality was	☐ 5	☐ 4	☐ 3	☐ 2	☐ 1
	**In the past 7 days…**	**Not at all**	**A little bit**	**Somewhat**	**Quite a bit**	**Very much**
Sleep116	My sleep was refreshing.	☐ 5	☐ 4	☐ 3	☐ 2	☐ 1
Sleep20	I had a problem with my sleep.....	☐ 1	☐ 2	☐ 3	☐ 4	☐ 5
Sleep44	I had difficulty falling asleep	☐ 1	☐ 2	☐ 3	☐ 4	☐ 5
	**Ability to Participate in Social Roles and Activities**	**Never**	**Rarely**	**Sometimes**	**Usually**	**Always**
SRPPER11_CaPS	I have trouble doing all of my regular leisure activities with others	☐ 5	☐ 4	☐ 3	☐ 2	☐ 1
SRPPER18_CaPS	I have trouble doing all of the family activities that I want to do...	☐ 5	☐ 4	☐ 3	☐ 2	☐ 1
SRPPER23_CaPS	I have trouble doing all of my usual work (include work at home)	☐ 5	☐ 4	☐ 3	☐ 2	☐ 1
SRPPER46_CaPS	I have trouble doing all of the activities with friends that I want to do....................................	☐ 5	☐ 4	☐ 3	☐ 2	☐ 1
	**Pain Interference In the past 7 days…**	**Not at all**	**A little bit**	**Somewhat**	**Quite a bit**	**Very much**
PAININ9	How much did pain interfere with your day to day activities? ...	☐ 1	☐ 2	☐ 3	☐ 4	☐ 5

PAININ22	How much did pain interfere with work around the home?........	☐ 1	☐ 2	☐ 3	☐ 4	☐ 5
PAININ31	How much did pain interfere with your ability to participate in social activities?.......................	☐ 1	☐ 2	☐ 3	☐ 4	☐ 5

	**Pain Interference**<br>**In the past 7 days…**	**Not at all**	**A little bit**	**Somewhat**	**Quite a bit**	**Very much**
PAININ34	How much did pain interfere with your household chores?.........	☐ 1	☐ 2	☐ 3	☐ 4	☐ 5

Pain Intensity
In the past 7 days…

Global07	How would you rate your pain on average?....................................	☐ 0<br>**No pain**	☐ 1	☐ 2	☐ 3	☐ 4	☐ 5	☐ 6	☐ 7	☐ 8	☐ 9	☐ 10<br>**Worst pain imaginable**

Supplement 3

Figure S1. Forest Plots for the Association of System-Based Symptoms with PROMIS-10 T Scores: (A) Global Physical Health and (B) Global Mental Health, Multivariable Linear Regression Adjusted for Covariables.

Table S1. Demographic and Clinical Characteristics of the CONTAIN-Extend Cohort by Enrollment Site

Table S2. Demographic and Clinical Characteristics of the CONTAIN-Extend Cohort vs. Non-Participants

Table S3. Adverse Events in the CONTAIN-Extend Cohort

Table S4. Symptoms in the CONTAIN-Extend Cohort by Randomization Arm at Baseline and Month 18

Table S5. Global Physical and Mental Health T-Scores from PROMIS Survey, breakdown by Clinical Variables

Table S6. Mean Scores of the PROMIS Items for Global Physical and Mental Health Assessment

Table S7. Inflammatory Markers in the CONTAIN-Extend Cohort at Baseline and Month 18

Figure S1. Forest Plots for the Association of System-Based Symptoms with PROMIS-10 T Scores: (A) Global Physical Health and (B) Global Mental Health, Multivariable Linear Regression Adjusted for Covariables.

A. Global Physical Health

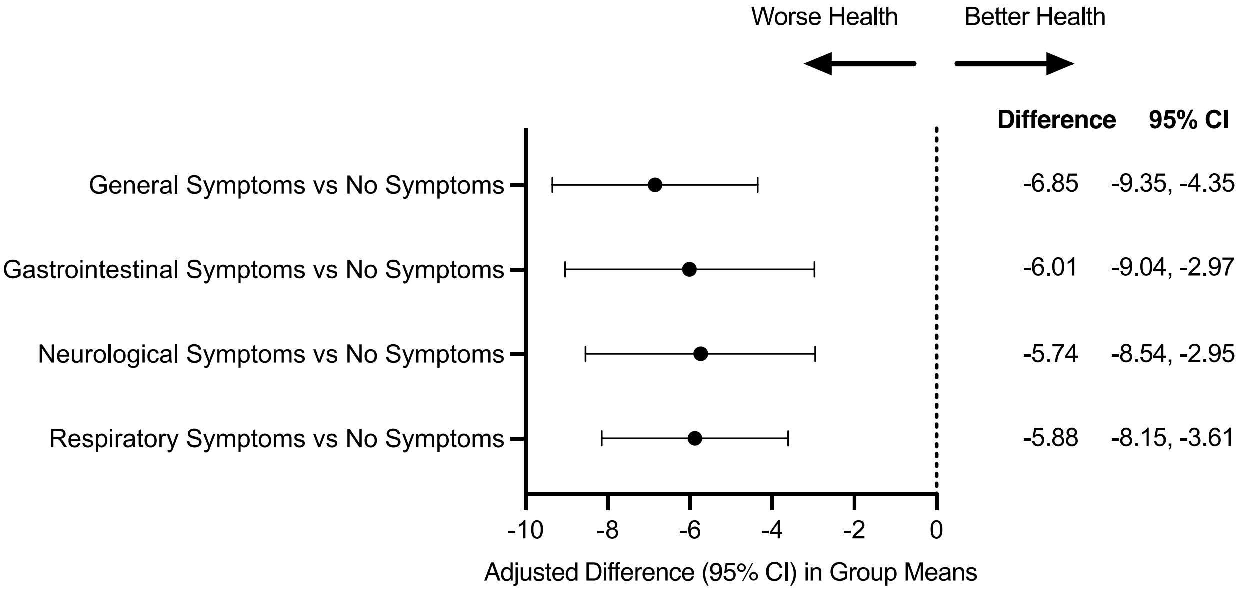

B. Global Mental Health

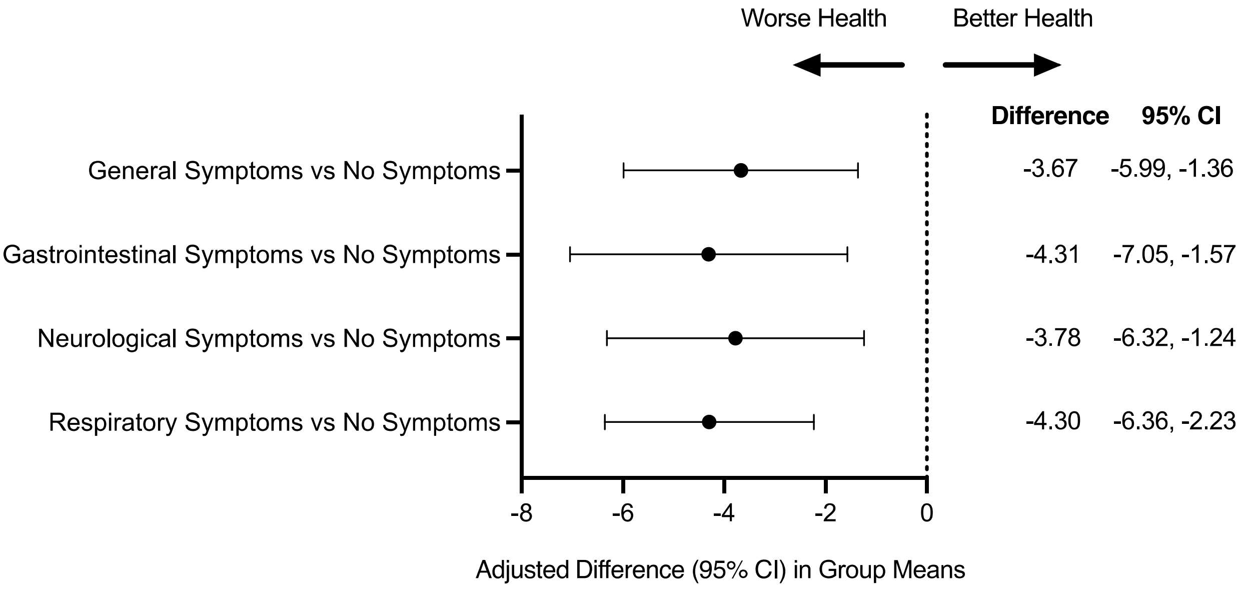

Table S1. Demographic and Clinical Characteristics of the CONTAIN-Extend Cohort by Enrollment Site

		New York		Connecticut	Florida	Texas	
	**Overall**	**NYU**	**Einstein**	**Yale**	**Miami**	**UT Houston**	**UT Tyler**
n	281	121	57	13	39	38	13
no. (%) consent among eligible	281/743 (37.8)	121/286 (42.3)	57/131 (43.5)	13/36 (36.1)	39/94 (41.5)	38/112 (33.9)	13/80 (16.3)
Age, median years (IQR)	59 (50-67)	60 (52-68)	59 (52-70)	58 (54-64)	61 (51-69.5)	53 (40-57.8)	60 (54-71)
Age, categorical, no. (%)							
<45	51 (18.1)	18 (14.9)	12 (21.1)	1 (7.7)	6 (15.4)	13 (34.2)	1 (7.7)
45-64	140 (49.8)	60 (49.6)	25 (43.9)	9 (69.2)	17 (43.6)	22 (57.9)	7 (53.8)
>65	90 (32.0)	43 (35.5)	20 (35.1)	3 (23.1)	16 (41.0)	3 (7.9)	5 (38.5)
Sex, no. (%)							
Male	156 (55.5)	71 (58.7)	30 (52.6)	8 (61.5)	24 (61.5)	17 (44.7)	6 (46.2)
Female	125 (44.5)	50 (41.3)	27 (47.4)	5 (38.5)	15 (38.5)	21 (55.3)	7 (53.8)
Race/Ethnicity[a], no. (%)							
Asian	20 (7.1)	18 (14.9)	2 (3.5)	0 (0.0)	0 (0.0)	0 (0.0)	0 (0.0)
Hispanic	120 (42.7)	27 (22.3)	31 (54.4)	1 (7.7)	27 (69.2)	34 (89.5)	0 (0.0)
Non-Hispanic Black	41 (14.6)	10 (8.3)	17 (29.8)	0 (0.0)	9 (23.1)	3 (7.9)	2 (15.4)
Non-Hispanic White	70 (24.9)	41 (33.9)	4 (7.0)	12 (92.3)	2 (5.1)	1 (2.6)	10 (76.9)
Other/Unknown[b]	30 (10.7)	25 (20.7)	3 (5.3)	0 (0.0)	1 (2.6)	0 (0.0)	1 (7.7)
BMI[c], median (IQR)	31.5 (26.6-36.5)	29.9 (25.8-36.0)	32.4 (26.4-36.1)	31.9 (28.4-38.4)	32.6 (27.9-38.5)	33.4 (29.0-38.5)	29.2 (24.5-38.2)
Enrollment quarter, no. (%)							
2020 Q2	40 (14.2)	33 (27.3)	7 (12.3)	0 (0.0)	0 (0.0)	0 (0.0)	0 (0.0)
2020 Q3 – 2021 Q5	241 (85.8)	88 (72.7)	50 (87.7)	13 (100.0)	39 (100.0)	38 (100.0)	13 (100.0)
WHO score at randomization, no. (%)							
5	220 (78.3)	95 (78.5)	53 (93.0)	10 (76.9)	24 (61.5)	28 (73.7)	10 (76.9)
6	61 (21.7)	26 (21.5)	4 (7.0)	3 (23.1)	15 (38.5)	10 (26.3)	3 (23.1)
Baseline spike IgG serostatus[d], no. (%)							
Negative	75 (26.7)	23 (19.0)	25 (43.9)	4 (30.8)	13 (33.3)	4 (10.5)	6 (46.2)
Positive	153 (54.4)	59 (48.8)	27 (47.4)	9 (69.2)	25 (64.1)	29 (76.3)	4 (30.8)
N/A	53 (18.9)	39 (32.2)	5 (8.8)	0 (0.0)	1 (2.6)	5 (13.2)	3 (23.1)
Comorbidities, no. (%)							
Pulmonary	21 (7.5)	6 (5.0)	8 (14.0)	0 (0.0)	3 (7.7)	1 (2.6)	3 (23.1)
Hypertension	158 (56.2)	63 (52.1)	35 (61.4)	7 (53.8)	27 (69.2)	18 (47.4)	8 (61.5)
Cardiovascular	100 (35.6)	47 (38.8)	17 (29.8)	7 (53.8)	14 (35.9)	12 (31.6)	3 (23.1)
Diabetes	82 (29.2)	33 (27.3)	17 (29.8)	2 (15.4)	12 (30.8)	13 (34.2)	5 (38.5)
Chronic Kidney Disease	27 (9.6)	11 (9.1)	8 (14.0)	0 (0.0)	4 (10.3)	4 (10.5)	0 (0.0)
Concurrent medications, no. (%)							
Remdesivir	171 (60.9)	43 (35.5)	44 (77.2)	12 (92.3)	35 (89.7)	30 (78.9)	7 (53.8)
Corticosteroids	232 (82.6)	90 (74.4)	47 (82.5)	12 (92.3)	38 (97.4)	33 (86.8)	12 (92.3)
Therapeutic anticoagulation	228 (81.1)	117 (96.7)	50 (87.7)	6 (46.2)	35 (89.7)	9 (23.7)	11 (84.6)
Months between randomization and 18-month visit, median (IQR)	17.2 (15.7-19.8)	16.0 (15.5-18.2)	16.3 (15.6-17.1)	18.5 (17.7-19.8)	21.9 (21.2-22.7)	19.4 (17.5-20.2)	19.2 (18.5-19.5)

COVID-19 vaccinated individuals[e], no. (%)	254 (90.4)	113 (93.4)	55 (96.5)	12 (92.3)	31 (79.5)	33 (86.8)	10 (76.9)

Abbreviations: BMI, body mass index; CCP, COVID-19 convalescent plasma; COVID-19, Coronavirus Disease 2019; IQR, interquartile range; N/A, not available; Q, quarter; WHO, World Health Organization

[a] Information on race and ethnic group was obtained from entries in the medical record, as reported by the patients.
[b] Other included mixed race, American Indian or Alaska Native, and Native Hawaiian or other Pacific Islander.
[c] BMI is calculated as weight in kilograms divided by height in meters squared.
[d] Defined as SARS-CoV-2 IgG titer greater than 1:100 using in-house full-length spike protein enzyme-linked immunosorbent assay.
[e] Includes all COVID-19 vaccines authorized or approved by the FDA: Pfizer-BioNTech, Moderna, and Johnson & Johnson's Janssen

Table S2. Demographic and Clinical Characteristics of the CONTAIN-Extend Cohort vs. Non-Participants

	Overall	CONTAIN-Extend	CONTAIN-Extend non-participants	Deceased
n	941	281	481	179
Age, median years (IQR)	63 (52-73)	59 (50-67)	63 (50-73)	71 (64-82)
Age, categorical, no. (%)				
<45	126 (13.4)	51 (18.1)	72 (15.0)	3 (1.7)
45-65	376 (40.0)	140 (49.8)	187 (38.9)	49 (27.4)
>65	439 (46.7)	90 (32.0)	222 (46.2)	127 (70.9)
Sex, no. (%)				
Male	556 (59.1)	156 (55.5)	280 (58.2)	120 (67.0)
Female	385 (40.9)	125 (44.5)	201 (41.8)	59 (33.0)
Race/Ethnicity[a], no. (%)				
Asian	71 (7.5)	17 (6.0)	34 (7.1)	20 (11.2)
Hispanic	373 (39.6)	116 (41.3)	182 (37.8)	75 (41.9)
Non-Hispanic Black	132 (14.0)	46 (16.4)	67 (13.9)	19 (10.6)
Non-Hispanic White	318 (33.8)	88 (31.3)	172 (35.8)	58 (32.4)
Other/Unknown[b]	47 (5.0)	14 (5.0)	26 (5.4)	7 (3.9)
BMI[c], median (IQR)	30.4 (26.1-36.1)	31.5 (26.6-36.5)	30.5 (26.4-36.3)	28.5 (24.5-33.9)
Enrollment quarter, no. (%)				
2020 Q2	170 (18.1)	40 (14.2)	73 (15.2)	57 (31.8)
2020 Q3 – 2021 Q5	771 (81.9)	241 (85.8)	408 (84.8)	122 (68.2)
WHO score at randomization, no. (%)				
5	673 (71.5)	220 (78.3)	377 (78.4)	76 (42.5)
6	268 (28.5)	61 (21.7)	104 (21.6)	103 (57.5)
Baseline spike IgG serostatus[d], no. (%)				
Negative	242 (25.7)	75 (26.7)	113 (23.5)	54 (30.2)
Positive	486 (51.6)	153 (54.4)	256 (53.2)	77 (43.0)
N/A	213 (22.6)	53 (18.9)	112 (23.3)	48 (26.8)
Comorbidities, no. (%)				
Pulmonary	97 (10.3)	21 (7.5)	57 (11.9)	19 (10.6)
Hypertension	571 (60.7)	158 (56.2)	270 (56.1)	143 (79.9)
Cardiovascular	404 (42.9)	100 (35.6)	198 (41.2)	106 (59.2)
Diabetes	332 (35.3)	82 (29.2)	178 (37.0)	72 (40.2)
Chronic Kidney Disease	99 (10.5)	27 (9.6)	45 (9.4)	27 (15.1)
Concurrent medications, no. (%)				
Remdesivir	538 (57.2)	171 (60.9)	285 (59.3)	82 (45.8)
Corticosteroids	722 (76.7)	232 (82.6)	371 (77.1)	119 (66.5)
Therapeutic anticoagulation	735 (78.1)	228 (81.1)	367 (76.3)	140 (78.2)

Abbreviations: BMI, body mass index; CCP, COVID-19 convalescent plasma; COVID-19, Coronavirus Disease 2019; IQR, interquartile range; N/A, not available; Q, quarter; WHO, World Health Organization

[a] Information on race and ethnic group was obtained from entries in the medical record, as reported by the patients.
[b] Other included mixed race, American Indian or Alaska Native, and Native Hawaiian or other Pacific Islander.
[c] BMI is calculated as weight in kilograms divided by height in meters squared.
[d] Defined as SARS-CoV-2 IgG titer greater than 1:100 using in-house full-length spike protein enzyme-linked immunosorbent assay.

Table S3. Adverse Events of the CONTAIN-Extend Cohort

	**Overall**	**Placebo**	**CCP**	***P*-value**
**n**	281	140	141	
**No. Adverse Events (%)**				
Chest pain	2 (0.7)	0 (0)	2 (1.4)	0.50
COVID-19 re-infection	12 (4.3)	6 (4.3)	6 (4.3)	>0.99
Exacerbation of asthma	3 (1.1)	0 (0)	3 (2.1)	0.25
Knee pain	2 (0.7)	1 (7.1)	1 (0.7)	>0.99
Nausea	2 (0.7)	0 (0)	2 (1.4)	0.50
Pneumonia	2 (0.7)	0 (0)	2 (1.4)	0.50
Shingles	3 (1.1)	1 (0.7)	2 (1.4)	>0.99
Sinusitis	2 (0.7)	2 (1.4)	0 (0)	0.25

Note: Adverse events occurred between the final 3-month visit of CONTAIN-RCT and time of month-18 CONTAIN-Extend visit.

Abbreviations: CCP, COVID-19 convalescent plasma; COVID-19, Coronavirus disease 2019
P-values, comparing the placebo and CCP groups, were estimated using a Fisher's exact test.

Table S4. Symptoms of the CONTAIN-Extend Cohort by Randomization Arm at Baseline and Month 18

	Baseline		Month 18	
	**Placebo**	**CCP**	**Placebo**	**CCP**
n	140	141	140	141
Any symptoms, no. (%)	127 (90.7)	127 (90.1)	79 (56.4)	77 (54.6)
General, no. (%)	51 (36.4)	59 (41.8)	41 (29.3)	40 (28.4)
Respiratory, no. (%)	123 (87.9)	120 (85.1)	51 (36.4)	55 (39.0)
Gastrointestinal, no. (%)	18 (12.9)	15 (10.6)	22 (15.7)	24 (17.0)
Neurological, no. (%)	17 (12.1)	22 (15.6)	34 (24.3)	28 (19.9)

Abbreviations: CCP, COVID-19 convalescent plasma

Table S5. Global Physical and Mental Health T-Scores from PROMIS Survey, breakdown by Clinical Variables

	Global physical health, mean (SD)	Global mental health, mean (SD)	n
**All**	46.4 (9.9)	50.0 (8.8)	277
**Baseline characteristics**			
Age, years			
<45	48.4 (10.8)	50.0 (9.9)	49
45-64	45.8 (9.7)	49.2 (8.4)	130
65	46.1 (9.7)	51.1 (8.6)	98
Sex			
Female	44.1 (10.4)	48.1 (8.7)	121
Male	48.2 (9.2)	51.5 (8.5)	156
Race/Ethnicity			
Asian	50.6 (7.7)	51.7 (7.4)	17
Hispanic	45.5 (10.1)	49.1 (8.1)	113
Non-Hispanic Black	43.5 (9.0)	49.0 (10.0)	47
Non-Hispanic White	47.6 (10.4)	51.0 (8.7)	88
Other/Unknown	50.6 (8.3)	53.3 (11.7)	12
Enrollment quarter			
2020 Q2	43.4 (10.4)	48.2 (7.8)	40
2020 Q3 – 2021 Q5	46.9 (9.8)	50.3 (8.9)	237
Baseline WHO scores			
5	46.3 (10.1)	50.0 (8.8)	217
6	46.4 (9.4)	50.6 (8.5)	60
Randomization arm			
Placebo	46.9 (9.4)	50.4 (8.9)	139
CCP	45.9 (10.4)	49.6 (8.7)	138
**18-month symptom assessment**			
Any symptoms			
No	50.4 (10.2)	52.3 (8.7)	116
Yes	43.5 (8.7)	48.4 (8.4)	161
General			
No	48.7 (9.5)	51.4 (8.5)	188
Yes	41.4 (9.0)	47.1 (8.6)	89
Respiratory			
No	48.8 (9.9)	51.8 (8.8)	169
Yes	42.6 (8.8)	47.3 (8.0)	108
Neurological			
No	47.8 (9.8)	51.0 (8.5)	210
Yes	42.0 (9.0)	46.9 (8.8)	67
Gastrointestinal			
No	47.5 (9.8)	50.9 (8.4)	231
Yes	40.4 (8.5)	45.6 (9.5)	46

Abbreviation: CCP, COVID-19 Convalescent Plasma; PROMIS, Patient-Reported Outcomes Measurement Information System; SD, standard deviation

Note: A higher score signifies better physical function or mental health. The US population’s average score is 50, with 10 points equivalent to 1 standard deviation.

Table S6. Mean Scores of the PROMIS Items for Global Physical and Mental Health Assessment

Item Stem, Scale 1-5	Mean (SD), n=277
**Global Physical Health**	
How would you rate your pain on average? (Global07)	3.7 (1.3)
To what extent are you able to carry out your everyday physical activities such as walking, climbing stairs, carrying groceries, or moving a chair (Global06)	3.8 (1.2)
In general, how would you rate your physical health? (Global03)	2.9 (0.9)
How would you rate for fatigue on average? (Global08r)	3.8 (0.9)
**Global Mental Health**	
In general, would you say your quality of life is (Global 02)	3.4 (0.9)
In general, how would you rate your mental health, including your mood and your ability to think? (Global04)	3.5 (1.0)
In general, how would you rate your satisfaction with your social activities and relationships? (Global05)	3.6 (0.9)
In the past 7 days, how often have you been bothered by emotional problems such as feeling anxious, depressed, or irritable? (Global10r)	4.1 (1.1)

Abbreviation: PROMIS, Patient-Reported Outcomes Measurement Information System; SD, standard deviation

Table S7. Inflammatory and Hematological Markers of the CONTAIN-Extend Cohort at Baseline and Month 18

	**Baseline**		**Month 18**	
	**Placebo**	**CCP**	**Placebo**	**CCP**
n	140	141	140	141
Absolute lymphocyte count, median (range), μ/L	0.76 (0, 12.1)	0.8 (0, 33.4)	1.9 (0.5, 24.2)	1.9 (0.07, 35.4)
Missing, no. (%)	8 (5.7)	4 (2.8)	27 (19.3)	23 (16.3)
Fibrinogen, median (range), mg/dL	648 (266, 1050)	630 (251, 1110)	380 (209, 663)	400 (208, 853)
Missing, no. (%)	36 (25.7)	34 (24.1)	31 (22.1)	33 (23.4)
Lactate dehydrogenase, median (range), U/L	411 (144, 1310)	356 (131, 1510)	192 (128, 337)	199 (108, 504)
Missing, no. (%)	30 (21.4)	25 (17.7)	31 (22.1)	33 (23.4)
Ferritin, median (range), ng/mL	751 (14.0, 19600)	688 (10.0, 13100)	95.5 (3.3-1510)	118 (4.0-2510)
Missing, no. (%)	5 (3.6)	9 (6.4)	28 (20.0)	31 (22.0)
C-reactive protein, median (range), mg/dL	84.0 (1.7, 424)	78.0 (0.3, 309)	2.0 (0.1, 36.4)	2.4 (0.1, 90.1)
Missing, no. (%)	5 (3.6)	10 (7.1)	35 (25.0)	35 (24.8)

SI conversion factors: To convert C-reactive protein to milligrams per liter, multiply by 10; ferritin to micrograms per liter, 1; fibrinogen to grams per liter, 0.01; lactate dehydrogenase to microkatals per liter, 0.0167; and lymphocytes to x10^9 per liter, 0.001.

Abbreviations: CCP, COVID-19 convalescent plasma

Supplement 4

Data Sharing Statement

Data

Data available: Yes
Data types: Deidentified participant data
How to access data: Send email to: CONTAINData@nyulangone.org
When available: at the time of manuscript publication.

Supporting Documents
Document types: None

Additional Information
Who can access the data: Qualified investigators.
Types of analyses: Primary outcome. Secondary outcome.
Mechanisms of data availability: Data request with description of proposed research will be reviewed by requesting investigators and link to download de-identified data from an open repository will be made available.

Supplement 5

The CONTAIN-Extend Study Group

Name	Role	Site
**CONTAIN Extend Coordinating Center**		
Judith S. Hochman, MD	CTSI PI	NYU Langone Health
Bruce N. Cronstein, MD	CTSI PI	NYU Langone Health
Deborah Keeling, MS	Project Finance	NYU Langone Health
Norka Rappoport, BS	Project Finance	NYU Langone Health
Jenna Saraga, MBA	Project Finance	NYU Langone Health
James Holahan, MS, MPH	Project Finance	NYU Langone Health
Mila B. Ortigoza, MD, PhD	Study PI, IND Holder	NYU Langone Health
Liise-anne Pirofski, MD	Study PI	Albert Einstein College of Medicine
Hyunah Yoon, MD	Study Co-PI	Albert Einstein College of Medicine
Caroline L. Sturm-Reganato, BA, BS, RN	Research Nurse Manager	NYU Langone Health
Gia F. Cobb, MA	Lead Study Project Manager	NYU Langone Health
Rakshit Andela, MBA, MScEng	Programmer	NYU Langone Health
Yousef Darwish, BS	Programmer	NYU Langone Health
Monica R. Taveras, BS	Study Data Manager	NYU Langone Health
Patrick S. Xin, MA, MS	Study Associate Data Manager	NYU Langone Health
Jeff LaFleur, MA	Biorepository Manager	Albert Einstein College of Medicine
Levi Cleare, BA	Biorepository/Laboratory technician	Albert Einstein College of Medicine
Keith S. Goldfeld, DrPH, MS, MPA	Lead Study Statistician	NYU Langone Health
Yi Li, MS	Study Statistician	NYU Langone Health

**NYU Langone Medical Center**		
Mila B. Ortigoza, MD, PhD	Site PI	NYU Langone Health (all locations)
Mary L. O'Keeffe, MD	Lead Co-Investigator	NYU Langone Health – Long Island
Gia F. Cobb, MA	Site Project Manager	NYU Langone Health
Caroline L. Sturm-Reganato, RN	Research Nurse Manager	NYU Langone Health
Fatema Z. Rahman, BS	Study Coordinator	NYU Langone Health
Adeyinka O. Ajayi, MD, MPH	Study Coordinator	NYU Langone Health
Sara L. Rodriguez, MBA	Regulatory Coordinator	NYU Langone Health
Eduardo Iturrate, MD, MSW	Site Associate Data Manager	NYU Langone Health
Jacqueline M. Gallagher, MSN, RN	Research Nurse	NYU Langone Health, Bellevue
Ololade E. Thomas, MPH BSN, RN,	Research Nurse	NYU Langone Health, Bellevue
Danibel Ramos, BSN, RN	Research Nurse	NYU Langone Health, Bellevue
Charlotte C. Fong, BS, RN	Research Nurse	NYU Langone Health, Bellevue
**Albert Einstein College of Medicine / Montefiore Medical Center**		
Liise-anne Pirofski, MD	Site PI	Albert Einstein College of Medicine
Hyunah Yoon, MD	Site Co-PI	Albert Einstein College of Medicine
Marla J. Keller, MD	Site PI	Albert Einstein College of Medicine
Andrea A. Asencio, BA	Site Coordinator	Albert Einstein College of Medicine
Isaiah Eke, MD MPH	Site Coordinator	Albert Einstein College of Medicine

James Castro, BE	Biorepository/Laboratory technician	Albert Einstein College of Medicine
Jidong Shan, PhD	Biorepository Manager	Albert Einstein College of Medicine
Alex Chalco, MS	Biorepository/Laboratory technician	Albert Einstein College of Medicine
Jeff LaFleur, MA	Biorepository Manager	Albert Einstein College of Medicine
Levi Cleare, BA	Biorepository/Laboratory technician	Albert Einstein College of Medicine
**Yale University Medical Center**		
Mahalia Desruisseaux, MD	Site PI	Yale University Medical Center
Grace M. Cortezzo, CPT, CMA	Site Data Manager & Site Coordinator	Yale University Medical Center
Erica Rocco, BS, CCRP	Regulatory Coordinator	Yale University Medical Center
Oscar Bate Akide Ndunge, PhD	Sub-Investigator	Yale University Medical Center
Catherine Parmelee, RN	Research Nurse	Yale University Medical Center
Gina Solomon, RN	Research Nurse	Yale University Medical Center
Staci Cahil, RN	Research Nurse	Yale University Medical Center
**University of Miami Consortium**		
Dushyantha T. Jayaweera, MD	Site PI	UHealth Tower, Jackson Memorial
Chin Chin Lee, MSPH, MS, BS	Site Project Manager & Regulatory Coordinator	UHealth Tower, Jackson Memorial
Daru L. Ransford, BA	Site Project Manager	UHealth Tower, Jackson Memorial
Deniz Dasmany, BS	Site Coordinator	UHealth Tower, Jackson Memorial
Andres Corona, BS	Data coordinator	UHealth Tower, Jackson Memorial
Kenia Moreno, RN, AS	Site coordinator	UHealth Tower, Jackson Memorial
Gledys L. Martinez, BA	Site coordinator	UHealth Tower, Jackson Memorial
Christopher Otero, BSN, RN	Research Nurse	UHealth Tower, Jackson Memorial
**Greater Texas COVID-19 Consortium (GTCC)**		
David D. McPherson, MD	Project PI	UTHealth-Houston
Luis Ostrosky-Zeichner, MD	Site PI	UTHealth-Houston
Bela Patel, MD	Site Co-PI	UTHealth-Houston
Masayuki Nigo, MD	Site Co-PI	UTHealth-Houston
Ryan M. Huebinger, MD	Site Co-PI	UTHealth-Houston
Goutham Dronavalli, MD	Site Co-PI	UTHealth-Houston
Carolyn Z. Grimes, DrPH	Laboratory Supervisor	UTHealth-Houston
Virginia E. Umana, BS	Site Project Manager	UTHealth-Houston
Maria D. Hernandez, MS	Laboratory technician	UTHealth-Houston
Laura E. Nielsen, BSN, RN, CCRN	Research Nurse	UTHealth-Houston
Taylor P. Stutz, BS	Site coordinator	UTHealth-Houston
Mehriban Mammadova, MPH	Site coordinator	UTHealth-Houston
Andrew N. Dentino, MD	Site PI	UT-Rio Grande Valley
Timothy R. Heath, MD	Site PI	UT-Rio Grande Valley
Jessica G. Martin, MD	Site Co-PI	UT-Rio Grande Valley
Fatimah O. Bello, MD	Site Co-PI	UT-Rio Grande Valley
Erik Hinojosa, BA	Site Coordinator	UT-Rio Grande Valley
Julie V. Philley, MD	Site PI	UT-Tyler
Megan S. Devine, MD	Site Co-PI	UT-Tyler
Rebekah L. Hibbard, BS, CCRC	Site Project Manager	UT-Tyler
Anne M. Ford, BA	Site Coordinator	UT-Tyler

Definition of Roles:

Role	Description
Project PI	Overarching PI named in grants
Project Finance	Oversees CONTAIN grants and distribution of funding to collaborating sites
Project Contracts	Oversees CONTAIN agreements and contracts with collaborating sites
Project Regulatory Coordinator	Oversees regulatory compliance at NYU site and CONTAIN liaison with FDA
Study PI	PI overseeing CONTAIN study at all sites
Site PI	PI lead for each site
Co-PI	Associate/Assistant PI for site or study
IND Holder	Named person in FDA's IND application
Study Statistician	Study statistician. Can be designated as "Lead"
Study Project Manager	PM overseeing CONTAIN study at all sites
Site Project Manager	PM overseeing site-specific research activities
Study Coordinator	Research coordinator or associate serving as adjunct role to study PM
Site Coordinator	Research coordinator or associate who conducts site-specific study activities, and serves as an adjunct role to site PMs/sub-Is. Can also be designated as "Lead Site Coordinator" if overseeing other coordinators.
Regulatory Coordinator	Research coordinator responsible for site-specific regulatory compliance
Study Data Manager	Blinded or unblinded data lead coordinator responsible for the data management Plan, data queries, and data cleanup.
Site Data Manager	Site-specific data manager
Study Associate Data Manager	DataCore directors advising CONTAIN CCC
Site Associate Data Manager	Site-specific DataCore director supporting NYU Langone Health Consortium
Programmer	Study programmer
Data Coordinator	Conducted site-specific data entries
Sub-Investigator	Conducts site-specific study activities with and without patient-facing contact. They make medical judgements and decisions regarding study subjects.
Research Nurse	Conducts site-specific study activities with and without patient-facing contact, and serves as an adjunct role to Sub-Is. Can also be designated as "Manager" if overseeing other RNs.
Biorepository/Laboratory Technician	Collect, store and/or analyze biospecimens in laboratory or Biorepository. Can also be designated as "Supervisor" or "Manager" if overseeing others.
